# Metastasis and Tumor Cell Migration of Solid Tumors

**DOI:** 10.3390/cancers13215576

**Published:** 2021-11-08

**Authors:** Jens Hoeppner, Peter Bronsert

**Affiliations:** 1 Department of Surgery, University Medical Center Schleswig-Holstein, UKSH Campus Lübeck, Ratzeburger Allee 160, 23538 Lübeck, Germany; 2 Institute for Surgical Pathology, Medical Center, Faculty of Medicine, University of Freiburg, Breisacher Strasse 115a, 79106 Freiburg, Germany

The developmental process of local and distant metastases represents the major and defining trait of malignant tumors, whereby tumor cells sustain the capability to migrate from the initial tumor site, seed, and grow at a location other than that of the initial tumor. To date, nearly 90% of all tumor-related deaths are caused by tumor metastasis. Tumor cell migration and invasion are crucial prognostic factors for tumor treatment response and patients’ long term survival. The mechanisms of metastatic spread, tumor cell migration and invasion are the focus of this Special Issue. Tumor cell migration and invasion take place at the tumor-host interface and are accompanied by a desmoplastic stroma reaction. This desmoplasia is a fibro-inflammatory process and an established feature of solid cancers such as pancreatic, lung, and breast cancer.

So-called “cancer-associated fibroblasts” are involved in the desmoplastic reaction. Cancer-associated fibroblasts are activated stromal cells that not only surround, supply, and protect the tumor but also provide a soil-and-seed platform for tumor metastases. In the near future, new biomarkers and improved antibody reagents, combined with new technical achievements, will lead to the identification of multiple subsets of cancer-associated fibroblasts in the context of various solid tumors. Consecutively, new targets in the tumor microenvironment for tumor treatment will be recognized.

Another important approach to explain tumor cell metastases is the epithelial-mesenchymal transition. The epithelial-mesenchymal transition is a fundamental model of embryogenesis in which polarized epithelial cells transform into motile epithelial cells with mesenchymal characteristics. This bi-directional embryonic cell differentiation and migration model, which allows for both epithelial-to-mesenchymal and, conversely, mesenchymal-to-epithelial cell differentiation, contains fundamental changes in the behavior and morphology of cells that affect cell migration and cell differentiation. As part of the epithelial-mesenchymal transition, tumor cells discard their morphological and molecular epithelial characteristics and adopt a mesenchymal subtype. A better comprehension of the role of epithelial-mesenchymal transition in solid tumors has demonstrated that it is not only important in the metastatic spread of cancer but also affects drug resistance and the acquisition of cancer stem cell properties. As epithelial-mesenchymal transition is involved in many crucial mechanisms of tumor progression, the interest in addressing epithelial-mesenchymal transition as a therapeutic target is strongly rising.

This Special Issue will highlight the role of different aspects of the process of epithelial-mesenchymal transition, tumor microenvironment, tumor-host interface interactions, tumor cell migration, and settlement of metastasis in solid tumor disease in order to improve our understanding of these complex interactions in human cancers. We are very happy to be able to support the distribution of knowledge and innovation in the research area of the tumor microenvironment with this Special Issue.

